# Graph theory in paediatric epilepsy: A systematic review

**DOI:** 10.1080/19585969.2022.2043128

**Published:** 2022-06-01

**Authors:** Raffaele Falsaperla, Giovanna Vitaliti, Simona Domenica Marino, Andrea Domenico Praticò, Janette Mailo, Michela Spatuzza, Maria Roberta Cilio, Rosario Foti, Martino Ruggieri

**Affiliations:** a Neonatal Intensive Care Unit, San Marco Hospital, University Hospital Policlinico “G. Rodolico-San Marco", Catania, Italy; b Unit of Pediatrics and Pediatric Emergency, University Hospital Policlinico “G. Rodolico-San Marco", Catania, Italy; c Department of Medical Sciences, Unit of Pediatrics, University of Ferrara, Ferrara, Italy; d Unit of Rare Diseases of the Nervous System in Childhood, Department of Clinical and Experimental Medicine, Section of Pediatrics and Child Neuropsychiatry, University of Catania, Catania, Italy; e Division of Pediatric Neurology, University of Alberta, Stollery Children’s Hospital, Edmonton, Alberta, Canada; f National Council of Research, Institute for Biomedical Research and Innovation (IRIB), Unit of Catania, Catania, Italy; g Institute for Experimental and Clinical Research, Catholic University of Leuven, Brussels, Belgium; h Department Chief of Rheumatology Unit, San Marco Hospital, University Hospital Policlinico “G. Rodolico-San Marco", Catania, Italy

**Keywords:** Graph theory, paediatric epilepsy, brain network, childhood

## Abstract

Graph theoretical studies have been designed to investigate network topologies during life. Network science and graph theory methods may contribute to a better understanding of brain function, both normal and abnormal, throughout developmental stages. The degree to which childhood epilepsies exert a significant effect on brain network organisation and cognition remains unclear. The hypothesis suggests that the formation of abnormal networks associated with epileptogenesis early in life causes a disruption in normal brain network development and cognition, reflecting abnormalities in later life. Neurological diseases with onset during critical stages of brain maturation, including childhood epilepsy, may threaten this orderly neurodevelopmental process. According to the hypothesis that the formation of abnormal networks associated with epileptogenesis in early life causes a disruption in normal brain network development, it is then mandatory to perform a proper examination of children with new-onset epilepsy early in the disease course and a deep study of their brain network organisation over time. In regards, graph theoretical analysis could add more information. In order to facilitate further development of graph theory in childhood, we performed a systematic review to describe its application in functional dynamic connectivity using electroencephalographic (EEG) analysis, focussing on paediatric epilepsy.

## Introduction

The first year of life marks the most rapid period of brain development on a micro- and macroscopic scale. On a cellular level, the brain grows an abundance of synaptic connections and those superfluous connections are pruned early in post-natal life (Flavell and Greenberg 2008). Moreover, long-distance axons start to be myelinated to improve signal transfer to the distant brain areas. This process continues until the fourth decade of life (Lebel et al. 2008; Tamnes et al. 2010; Yap et al. 2011). Literature data show that such maturational processes lead to network topologies with brain functional networks shifting from more random towards a more ordered configuration (Smit et al. 2010; Boersma et al. 2011; Smit et al. 2012).

Graph theoretical studies have been designed to investigate network topologies during life. Network science and graph theory methods may contribute to a better understanding of brain function, both normal and abnormal, throughout developmental stages (Bullmore and Sporns 2009; Griffa et al. 2013). In particular, they could contribute to connecting brain structure to its function, clarify the link between structural changes and functional derangement, and explore how cognitive processes emerge from their morphological substrates (Sporns et al. 2005). Several research groups have recently focussed on brain functional analysis implementing the graph theory applications Sporns and Zwi 2004; Stam and Reijneveld 2007; Fallani et al. 2007; He et al. 2007; Rubinov and Sporns 2010; Vecchio et al. 2014a; 2014b; Vecchio et al. 2015a; 2015b; Vecchio et al. 2016a; 2016b), using a variety of methodological approaches and datasets. Particularly in epilepsy, the graph theory could add to a fast-growing research field focussed on network abnormalities in epileptogenesis, seizure propagation and refractoriness. In order to facilitate further development of graph theory in this new field, we performed a systematic review to describe its application in functional dynamic connectivity using electroencephalographic (EEG) analysis, focussing on paediatric epilepsy.

## Methods

**Figure 1.**
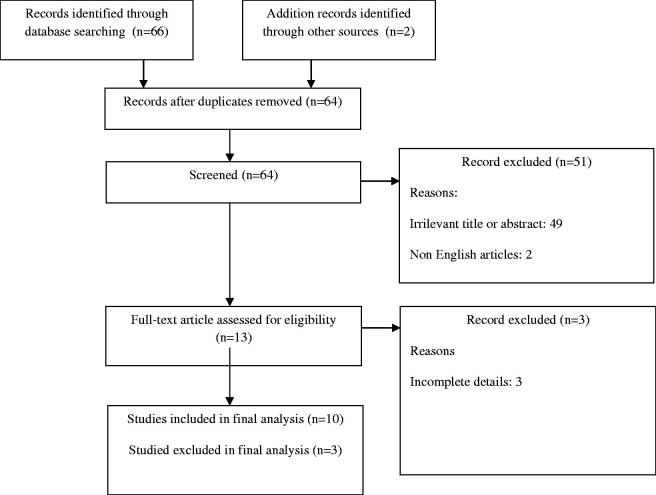
Flowchart of the literature search process.

This review was performed in accordance to the “preferred reporting items for systematic review” (PRISMA) (Figure 1), and “the assessment of the methodological quality of systematic reviews” (AMSTAR-2) guidelines (Moher et al. 2009; Shea et al. 2017). We carried out a systematic review of available literature on several medical electronic databases (including Cochrane Library, Medline, PubMed Central, Scopus and Web of Science). We used the following keywords: “graph theory” OR “brain network connections” OR “EEG graph theoretical studies,” AND “epilepsy,” AND/OR “EEG application,” AND/OR "computational EEG network." The studies considered were published in English from inception to December 2020. Studies from the reference lists of relevant articles retrieved were also analysed.

### Eligibility criteria

We selected studies performed on children with epilepsy (age ranging from 1 month to 18 years), in whom graph theory was applied for both diagnostic and predictive purposes, i.e., to evaluate the recurrence of seizures. Only the studies in which graph theory was applied to the evaluation of the surface EEG were selected.

### Exclusion criteria

Review articles, case report, case series and studies with incomplete details or from which the required data could not be extracted were excluded from our revision.

### Study selection

One author (S.D.M.) searched all the databases according to the search strategies, and duplicate studies were excluded. Three authors (R.F, S.D.M, G.V.)) independently screened titles, abstracts and full texts to identify eligible studies. All reasons for excluding studies were recorded.

### Quality appraisal

Two authors (S.D.M. and G.V.) independently assessed the risk-of-bias of the selected studies according to the 11-item instrument recommended by the Agency for Healthcare Research and Quality (AHRQ) for cross-sectional studies (Chang et al. 2012). For each item, we answered “yes,” “no,” or “unclear,” and studies that received a score of seven stars or more were considered high-quality papers. Disagreements between the two independent reviewers (S.D.M. and G.V.) were discussed and resolved by a third reviewer (R.F.). We used the Cohen kappa statistic to calculate the level of disagreement and an achieved good agreement was considered for value >80% (Sim and Wright 2005). (Table 1).

### Results

64 titles and abstracts were screened, 51 were excluded because they did not focus on the topic; 13 full texts were read and 3 were excluded for incomplete details. In this review, 10 articles were included (Figure 1).

## The graph theory approach

The human brain can be thought of as a complex of interconnected networks. Research studies on the complexities of brain networks are relatively new but rapidly expanding. Most studies refer to the connection matrix of the human brain as the human "connectome" (Gaal et al. 2010; Vecchio et al. 2017). Graph theory applied to the human brain is essentially a mathematical representation of the real brain architecture with a set of nodes (vertices), representing brain regions, and links (edges), representing anatomical, functional, or effective connections, interposed between them (Rubinov and Sporns 2010).

In this theory, the network is composed of a matrix, in which each row represents a node, and each column represents the relationship between nodes in the network. Links between nodes can be weighted or unweighted. Weighted links represent the density, size and coherence of anatomical tracts in anatomical networks, symbolising the strength of correlation or causal interactions in functional networks (Vecchio et al. 2017). Unweighted (binary) networks are created by applying a threshold to a weighted network, in which links indicate the presence or absence of any connection (Vecchio et al. 2017). Although literature data often use unweighted networks, interest in weighted network analysis is increasing because of more specific information provided by the weighted connections (Telesford et al. 2011).

Algorithms based on network connections provide parameters that define the global organisation of the brain and its alterations (Griffa et al. 2013). Researchers have applied the graph theory to EEG data analysis in order to investigate the brain network organisation changes associated with ageing (Vecchio et al. 2014a; 2014b). It was observed that both measures of local segregation (clustering as an index of local interconnections and network segregation) and global integration (path length as an index of information transfer efficiency) could discriminate cortical network features, which represent the confine between physiological and pathological neurodegenerative brain ageing. This new approach resulted in the development of functional connectivity models with the aim to clarify whether there is an optimal balance between global integration and local independence as a favourable condition for information processing (Gaal et al. 2010).

## Parameters derived by graph theory analysis

Recent literature data have applied graph theory to brain imaging with promising results as an interpretable and generalisable way to draft model brain networks (Rubinov and Sporns 2010). In this theory, a graph is a mathematical construct to represent a model in which the relationship between objects is described. These objects are named *vertices* and their interconnecting links are called *edges* (Rubinov and Sporns 2010).

In this brain network, regions of interest (ROIs) can be drafted by these vertices in a graph, and some measures of connectivity between ROIs, are represented by the edges. One important advantage of this model is that simple, numerical summary descriptors of graph organisation can be derived, describing the graph structure with a topology graph in terms of the whole network (Rubinov and Sporns 2010; Telesford et al. 2011).

The metrics mostly used to describe this graph are named: *characteristic path length* (that measures how easy to easy it is to traverse the whole graph), *clustering coefficient* (a measure of local connectivity), and *small-worldness* (that represents the state of being highly clustered, even having a short average path) (Rubinov and Sporns 2010).

These measures provide a way to characterise the underlying structural and functional brain networks, allowing comparison across subjects and time. In a graph theory analysis, segregation refers to the degree to which network elements form separate clusters and reflect a clustering coefficient (C) (Rubinov and Sporns 2010), while integration refers to the capacity of network exchange information to become interconnected, and it is defined by the characteristic path length (L) coefficient (Rubinov and Sporns 2010).

**Figure 2.**
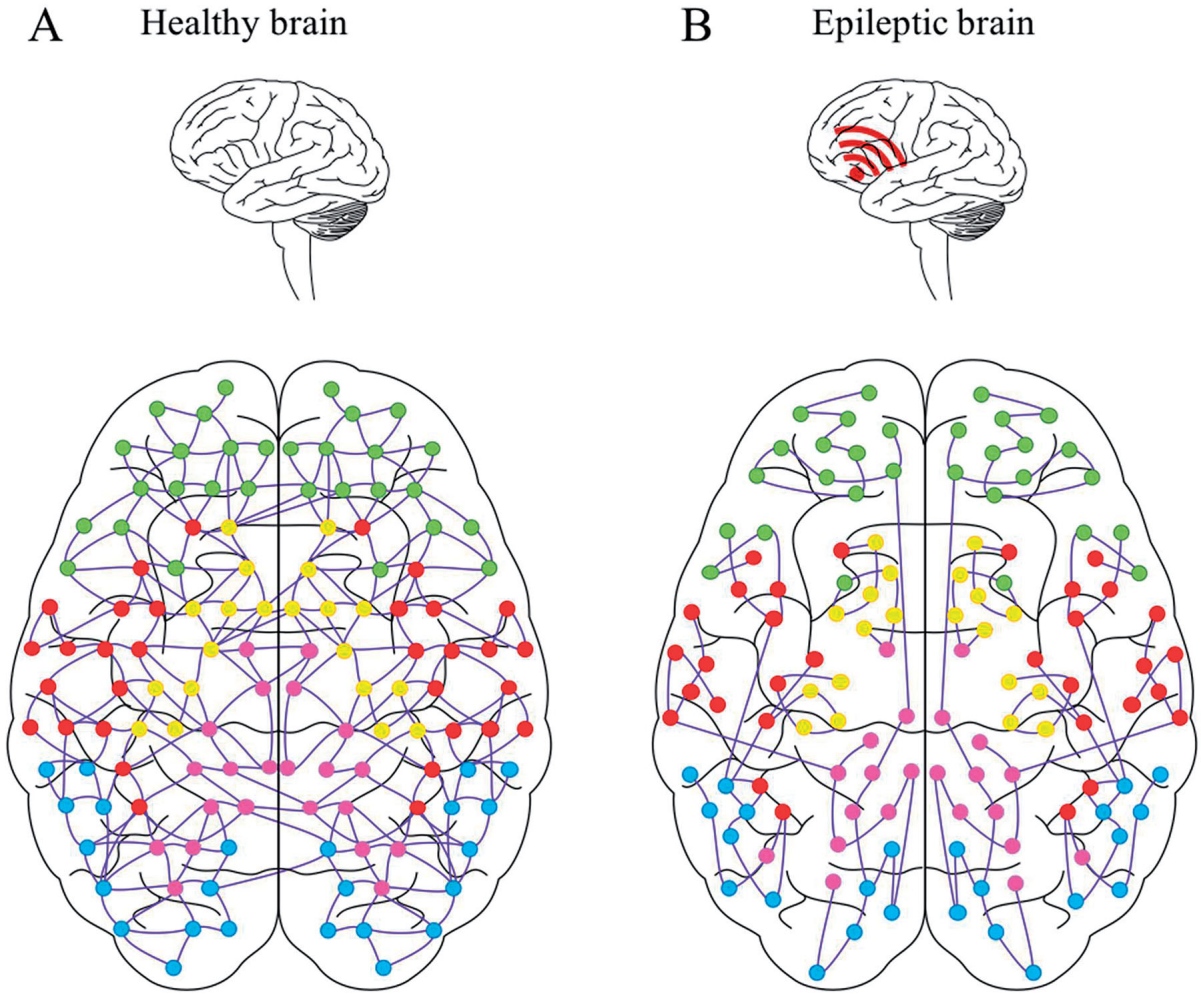
Visual example of the connectome in healthy brain (A) vs epileptic brain (B).

The mean clustering coefficient is computed for all the studied nodes of the graph and then averaged (Rubinov and Sporns 2010). This allows a measure of the tendency of network elements to form local clusters (de Haan et al. 2009). According to Figure 2a healthy brain is represented, while in Figure 2b the epileptic brain is designed.

Starting with the definition of the L coefficient (Rubinov and Sporns 2010), the weighted characteristic path length (defined as Lw) represents the shortest weighted path length between two nodes. (Rubinov and Sporns 2010).

The Small-world (SW) parameter represents the ratio between normalised C and L – Cw and Lw – with respect to the frequency bands (Rubinov and Sporns 2010). The SW coefficient describes the balance between the global integration of a network and local connectivity. SW organisation is an intermediate step between random networks -the short overall path length associated with a low level of local clustering - and regular networks - the high level of clustering which is associated with a long L coefficient (Vecchio et al. 2014b). This means that most nodes maintain a few direct connections and are linked by a relatively few.

## Application of graph theory to EEG for the study of childhood epilepsy

Studies focussing on graph theory application to EEG analysis to study the physiological brain maturation (Vecchio et al. 2017). have been expanding. Boersma and colleagues investigated the records of resting-state eyes-closed EEG of young children (5–7 years). The graphs were weighted using synchronisation likelihood (SL). The authors showed an increase in average C and L coefficients with age and suggested that there is a shift from random to more organised SW functional networks during brain maturation in childhood (Boersma et al. 2011).

Micheloyannis et al published a study of SL in the EEG of children (8–12 years) and young adult students (21–26 years). They found that beta and gamma values of the C coefficient were higher in children than in young adults. The authors also found that SW in the beta band was significantly higher in children than in young adults. They concluded that in children there is a higher synchronisation of fast frequencies, reflecting brain maturational processes (Micheloyannis et al. 2009).

Brain networks have been shown to change their dynamic state, switching between rest and movement, cognitive and behavioural tasks, and wakefulness and sleep. In patients with epilepsy, brain networks may show the transient occurrence of paroxysmal firing within neuronal groups, which could evolve into a seizure over time (Vecchio et al. 2017). In this context, characterisation of neural networks in epilepsy has become more relevant since focal epilepsy could be related to abnormal functioning of specific brain networks without any structural damage (Vecchio et al. 2017).

Seizures are considered the result of an imbalance between excitatory and inhibitory signals, leading to a hyperexcitable state in which the abnormal rhythms of neural firing cannot be sufficiently controlled by physiological inhibitory mechanisms. This imbalance would then generate a paroxysmal depolarisation shift (Stafstrom and Carmant 2015).

The relevance of graph theory application to EEG relies on the assumption that seizures are caused by a progressive hyper-synchronization of the firing of a critical mass of neurons, implying that a single neuron cannot cause a seizure, and the involvement of a population of cells involved in networks of neuronal assemblies is necessary (Engel et al. 2013; Mei et al. 2018).

In the human brain, optimal networks among structures with effective connectivity are believed essential for proper information processing (Vecchio et al. 2017). It seems that an association exists between pathological changes in connectivity, network structures and functional abnormalities of the brain.

The degree to which childhood epilepsies exert a significant effect on brain network organisation and cognition remains unclear. The hypothesis suggests that the formation of abnormal networks associated with epileptogenesis early in life causes a disruption in normal brain network development and cognition (Berman et al. 2010; Cainelli et al. 2020). Nevertheless, the pattern, mechanisms, and timing by which the organisation of structural networks in the developing brain may be altered are still unknown. Studying the new onset of childhood epilepsies and the associated brain network organisation over time may reveal more information.

Given that EEG is a representation of the synchronous activity of neurons arranged perpendicular to the surface of the cerebral cortex, this methodology does not allow a direct measure of subcortical and white matter interconnections. This might impact the diagnosis of seizures arising from subcortical areas, such as autonomic seizures occurring in the prelimbic cortex and the surrounding areas (Cainelli et al. 2020).

Nevertheless, several EEG algorithms offer an interesting insight into how cortical areas work together within the networks; and given their high temporal resolution, the link with the resulting cognitive processes will become clearer over time (Cainelli et al. 2020).

Among newly developed analysis techniques, graph theory has shown promising results given its possibility to provide information about global and local levels of the brain organisation. All these graph metrics provide evidence of both integration (e.g., strength) and segregation (e.g., clustering) properties of the network. An emergent property of many complex networks in nature is the “small-world” topology, which is random (i.e., each node is randomly connected to all the other nodes) and interconnected between regular (i.e., each node is linked only to its neighbors) graph topologies. Small-world characterises graphs with dense local clustering and relatively few long-range connections, which is an interesting property because it can globally account for both distributed (integrated) and specialised (segregated) information processing.

It has been suggested that the prevalence of small-world networks in biological systems may reflect the evolutionary advantage of greater sustainability of system performance under any kind of perturbations^38^. The advantage of EEG theoretical analysis with respect to brain images is that, although brain imaging may reveal both structure and function, EEG provides complementary information on the pathological brain area with much faster time scales (milli- seconds) combined with a relatively easier assessment compared to neuroimaging (Salvador et al. 2005).

Nevertheless, the EEG may be technically challenging in paediatric patients lacking cooperation, or in the intensive care setting with an abundance of environmental artefacts^37^. Furthermore, in children, a smaller head surface for electrode placement (both due to the presence of medical equipment and to the smaller head circumference) reduces the possibility of measuring many scalp sites. Besides all these limitations, the EEG still represents a very useful tool to study the developmental trajectories in childhood. EEG offers a unique window into the brain function during the early phases, which parallel basic science studies of neurochemical and histological processes associated with neuroimaging studies (Suppiej et al. 2012).

## Discussion

In our study, we found that the papers we included mainly focussed on two potential advantages of graph theory in childhood epilepsy. In regards, it seems that graph theory has not only shown the ability to diagnose epileptic foci distant to the main one known to be responsible for that kind of epilepsy but also the diagnostic method is able to predict the onset of epilepsy when the main foci have not started its hypersynchronous activity, by studying network connections with other relevant areas that are linked with the main brain area responsible for that kind of epilepsy.

### Diagnostic value of graph theory in childhood epilepsy

To increase the knowledge of seizure generation, much research has been done on seizure dynamics and changes in synchronisation between cortical areas (Ponten et al. 2009). Synchronisation of neural activity within the brain is essential for information processing, but may also be an important factor in seizure dynamics (Uhlhaas and Singer 2006). Changes in synchronisation during and before seizures have been investigated in model and functional network studies. Seizures are often described as ‘hypersynchronous states’, but literature data showed that this description is an oversimplification of the synchronisation process during seizures caused by various aetiologies (Wendling et al. 2005; Guye et al. 2006; Schindler et al. 2007) (Figure 2a,b).

Several studies have been performed on paediatric patients with epilepsy to evaluate functional neural connections, and all of them used graph theory analyses to explain this hypothesis. In 2009, Ponten et al. studied whether neural networks in weighted and unweighted networks can be detectable in generalised absence seizures recorded with surface EEG. The authors retrospectively selected EEG recordings of 11 children with absence seizures. The functional neural networks were studied by calculating both central coherence and SL between 21 EEG signals. From both weighted and unweighted networks, the C and L coefficients were computed and compared to 500 random networks. The authors then made a comparison between the ictal and the pre-ictal network structures. They found that during absence seizures there was an increase of synchronisation in all frequency bands, most clearly evidenced in the SL- based networks. Contextually, the functional network topology changed towards a more ordered pattern, with an increase of C/C-s and L/L-s. The authors, therefore, concluded supporting the hypothesis of functional neural network changes during absence seizures. The network became more regularised in weighted and unweighted analyses, when compared to the more randomised pre-ictal network configuration (Ponten et al. 2009).

Later, in 2015, Adebimpe et al investigated the functional connectivity and brain network properties of patients affected by benign childhood epilepsy with centrotemporal spikes using graph theory. Benign childhood epilepsy with centrotemporal spikes is considered the most common form of idiopathic epilepsy in young children under the age of 16 years. The authors collected high-density EEG data from patients and controls in resting state with eyes closed. Data were pre-processed and spike and spike-free segments were selected for their analysis. Therefore, phase-locking value was calculated for all paired combinations of channels and for five frequency bands (δ, θ, α, β_1_ and β_2_). The authors computed the degree and small-world parameters—C and L coefficients—and compared the conditions of the epileptic patients to controls. They found a higher degree at epileptic zones during interictal epileptic spikes (IES) in all frequency bands. In both patient conditions, connection at the occipital and right frontal regions close to the epileptic zone in the α band was reduced. The SW features (high C and short L) were deviated in epileptic patients compared to controls. A change from an ordered network in the δ band to a more randomly organised network in the α band was observed in epileptic patients compared to healthy controls. The authors then concluded that the benign epileptic brain network is disrupted not only at the epileptic zone but also in other brain areas especially frontal regions (Adebimpe et al. 2015).

A similar study was performed in 2016 by Chany et al. in patients affected by juvenile myoclonic epilepsy (JME). The authors compared the network properties of periods of spike-waves discharges and baseline activity using graph theory. They collected the EEG data of 11 patients with JME. Functional cortical networks during SWD and baseline periods were estimated by calculating the coherence between all possible electrode pairs in the δ, θ, α, β1 and β2, and ϒ bands. Graph theoretical measures (including characteristic L, nodal degree, C coefficient, and SW index) were then used to study the characteristics of epileptic networks in JME. The authors also assessed short- and long-range connections between spike-waves discharges and baseline networks. They found that compared to baseline, increased coherence was observed during spike-waves discharges in all frequency bands. The nodal degree of the spike-waves discharges network, particularly in the frontal region, was significantly higher compared to the baseline. The C coefficient and SW index were significantly lower in the theta and beta bands of the spike-waves discharges versus the baseline network, but the characteristic path length did not differ among epileptic and controls networks. Long-range connections were increased during spike-waves discharges, particularly between posterior and frontal brain regions. The authors then concluded that spike-waves discharges in JME are associated with increased local (particularly in the frontal region) connectivity. Furthermore, the spike-waves discharges network was associated with reduced small-worldness and increased long-range connections, which may impair information processing during spike-waves discharges (Lee et al 2016).

Finally, in 2016, Van Diessen et al. performed a prospective study on 89 drug-naive children with newly diagnosed focal or generalised epilepsies and 179 controls. Their patients underwent interictal EEG recordings at the first consultation and then studied for analysis of brain networks. Conventional network metrics and minimum spanning trees were computed to evaluate topological network differences, including segregation, integration, and a hub measure (including centrality). The authors found that network alterations between groups were only identified by minimum spanning tree metrics and most pronounced in the δ band. In this band, they observed a loss of network integration and a significantly lower betweenness centrality in children with focal epilepsy compared to controls without epilepsy (p < 0.01). The authors moreover found a reversed group difference in the upper α band. In generalised epilepsy, the network topology was relatively spared. The authors, therefore, concluded that interictal network alterations were only detectable in the minimum spanning tree metrics, and are already present at the early stages of focal epilepsy. They suggested that these alterations seem to be subtle at early stages, to consequently aggravating later as a result of persisting seizures (van Diessen et al. 2016).

Taken together, these studies confirm the diagnostic importance of graph theory, above all to detect epileptic foci distant to the original one and to improve the knowledge of network functions and connectivity in the epileptic brain. Nevertheless, different parameters have been considered between studies, and further literature data are mandatory to establish which protocols and parameters have to be considered for better diagnostic specificity and sensibility.

### Graph theory for seizure prediction in childhood epilepsy

Feature-based neurophysiological techniques tend to oversimplify in the diagnostic process the fact that the human brain involves a complex web of neuronal interconnectivity and discrete anatomical regions that function together to generate brain activity (Lowe et al. 1998). This underlying theory on brain infrastructure considers that solutions to the diagnosis of epilepsy need to consider the whole-brain functional connectivity network (FCN). Thus, this connectivity network seeks to define a pattern of cross-correlation between discrete functionally characterised brain regions to give further statistical importance to anatomical connectivity and then determine inter-regional neurophysiological inferences (Lowe et al. 1998, Seth 2010). This allows constructing a model of connectivity not only focussed on the diagnosis of epileptic events but also able to predict these events when an epileptic area has not still identified.

In regards, Sargolzaei et al, in 2014, performed a study on paediatric patients to establish a new data-driven approach to brain functional connectivity networks using scalp EEG recordings for classifying paediatric subjects with epilepsy from paediatric controls. The authors studied 16 children, 9 affected by paediatric epilepsy (PE) and 7 controls (PC). In this study, scalp EEG was obtained with varying sampling frequencies of 200 Hz, 500 Hz and 512 Hz from control subjects (4 males and 3 females) and subjects with epilepsy (5 males and 4 females). The EEG traces were recorded using the 10–20 electrode placement system with a referential montage. In the same patients, the authors performed a study of the "Functional Connectivity Network Construction" (FCNs) by graph theory analyses. The study of functional connectivity was based on the association among the EEG recordings across the brain cortical regions. The system used topological features extracted from a graph corresponding to the FCN as predictive variables, and it categorised the subjects into control and epileptic groups by defining the best model to predict the diagnosis. The authors found significant changes in the brain FCNs in epileptic patients. They demonstrated that through connectivity maps, the physiological manifestations of abnormal cortical excitability that underlie epilepsy could infer the occurrence of high-risk level (epileptic children) and low-risk level (control children) leading to follow-up procedures. The authors, therefore, proposed to use key parameters through connectivity maps in order to classify EEG files into epileptic and non-epileptic files (Sargolzaei et al. 2015).

The same research group, in 2015, published a prospective study on 18 subjects (11 with paediatric epilepsy (PE), and 7 controls (PC)) to introduce a new time-varying approach for constructing FCNs, implemented by moving a window with overlap to split the EEG signals into a total of 445 multi-channel EEG segments (91 for PC and 345 for PE). The aim was to study the hypothetical functional connectivity strengths among EEG channels. FCNs were then mapped into undirected graphs and extracted by graph theory-based features. The authors moreover used an unsupervised labelling technique based on the Gaussian mixtures model (GMM) to delineate the PE group from the PC group.

The authors found a statistically significant difference (p < 0.0001) between the mean FCNs of PC and PE groups. The system was able to diagnose PE patients with an accuracy of 88.8% and 81.8% of sensitivity, and 100% of specificity purely based on the exploration of networks between cortical areas and without a priori knowledge of the diagnosis. The authors, therefore, concluded that the system could allow a diagnosis and prediction of epilepsy without needing long EEG recording sessions and time-consuming visual inspection as conventionally employed (Sargolzaei et al. 2015).

In 2017, Lee et al. retrospectively compared the graph-theoretical characteristics of spike-and-wave discharge (SWD) network topology in JME patients, aiming to further elucidate the mechanisms underlying SWD. The authors first evaluated nodal degree reported as a key feature in generalised epilepsy. Second, they studied the graph-theoretical measures in different frequency bands, including C, L, and SW. These metrics, above all C and L, are related to the local and global efficiency of the network respectively. Finally, the regional connectivity of the 2 different states was studied and compared (Lee et al. 2017).

These data were obtained from 11 patients with Juvenile Myoclonic Epilepsy (JME), described as an idiopathic generalised epileptic syndrome, characterised by SWD EEG waveforms. The authors studied functional cortical networks during SWD and baseline periods by calculating the coherence between all electrode pairs in different frequency bands (delta, theta, alpha, beta, and gamma). Graph theoretical results, including nodal degree, C, L, and SW index, were then used to study the epileptic networks. (Lee et al. 2017).

The authors also assessed short- and long-range connections between baseline networks and SWD. They observed that compared to baseline, increased coherence was present during SWD in all EEG bands. The nodal degree of SWD was higher in the frontal region when compared to the baseline network. SW index and C were significantly lower in the beta and theta bands during SWD compared to the baseline network, but L did not differ among networks (Lee et al. 2017). Long-range connections were increased during SWD, prevalently between the posterior cortical areas and the frontal region (Lee et al. 2017). The authors, therefore, concluded that the SWD network in JME was associated with increased local (mainly in the frontal region) connectivity. Furthermore, the SWD network was associated with increased long-range connectivity and SW that may impair information processing during the discharges (Lee et al. 2017).

Later, in 2019, Luckett et al. proposed a study on graph theoretical analysis of seizure onset observed from minimally invasive scalp EEG. The approach considered the brain as a complex nonlinear dynamical system whose state could be derived through time-delay embedding of the EEG data and characterised to determine the change in brain dynamics according to the preictal state.

In this study, the authors included 41 temporal lobe epilepsy patients and 20 controls. Ages ranged from 4 to 57 years old. Among the included patients, 10 with epilepsy were under 18 years of age, and 3 paediatric patients were controls. The authors found a significant trend of normalised dissimilarity over time that indicates a departure from the norm, and thus a change in state. These methods showed high sensitivity (90-100%) and specificity (90%) on 241 h of scalp EEG training data, and sensitivity and specificity of 70%-90% on test data. The authors concluded that this method was able to target phase-space graph spectra as biomarkers for seizure prediction, correlate historical degrees of change in spectra, and make accurate predictions of seizure onset (Luckett et al. 2019).

In the same year, Davis et al. published a study to identify whether abnormal EEG connectivity was present before the onset of epileptic spasms (ES) in infants with tuberous sclerosis complex (TSC). The authors prospectively collected Scalp EEG recordings in infants with TSC in the first year of life. They then compared the earliest recorded EEG from infants prior to ES onset (n = 16) and from infants who did not develop ES (n = 28). All patients underwent a record of five minutes of stage II or quiet sleep into canonical EEG frequency bands. Mutual information values between each pair of EEG channels were compared directly and used as a weighted graph to calculate graph measures of modularity, global efficiency, characteristic L, and average C coefficient. The authors found that infants who later developed ES showed increased EEG connectivity in sleep. This group presented higher mutual information values between most EEG channels in all frequency bands adjusted for age. Infants who later developed ES had higher average clustering coefficients and global efficiency, shorter characteristic path lengths, and lower modularity across most frequency bands adjusted for age. The authors, therefore, suggested that infants who went on to develop ES had increased local and long-range EEG connectivity with less segregation of graph regions into distinct modules. This overconnectivity may reflect progressive pathologic network synchronisation culminating in generalised ES (Davis et al. 2019).

Finally, in 2020, Bomela et al. published a study on 23 paediatric patients with epilepsy. They performed detection and prediction of epileptic seizures with EEG by a novel dynamic learning method that first infers a time-varying network constituted by multivariate EEG signals, representing the overall dynamics of the brain network, and subsequently quantifies its topological property using graph theory. The computational results for a realistic scalp EEG database showed a detection rate of 93.6% and a false positive rate of 0.16 per hour (FP/h); furthermore, this method could observe potential pre-seizure phenomena in some cases (Bomela et al. 2020).

Taken together, all these studies show an important characteristic of graph theory, involved not only in a deep diagnosis of paediatric epilepsy but also in its prediction, allowing to avoid long EEG recording sessions and time-consuming visual inspection when the diagnosis is difficult. This would allow detecting seizures in a preictal stage, with a better study of all those cortical areas involved (Falsaperla et al. 2021). Nevertheless, further studies are mandatory to state the timing for prediction and its eventual employment for therapeutic purposes.

## Conclusions

Literature data have confirmed the utility and efficiency of graph theory application to study epilepsy at all ages. Epileptic foci can be distant from the area involved in the clinical manifestation of seizures, and therefore the integration of EEG with graph theory may be an important step in the identification of the epileptogenic areas not evident in a simple EEG analysis.

Interestingly, there are substantial differences between adult and childhood epilepsy when studied by graph theory. A more profound brain network reorganisation is evident in the developing brain. Children with new-onset epilepsy also show a reduced optimal topological structural organisation with a bias towards curtailed global integration and enhanced network segregation. Importantly, these properties are evident at very early stages in the course of epilepsy and are clearly not a consequence of epilepsy chronicity. Nevertheless, we have to consider that in children epileptic "syndromes" are different from adults. Therefore, studies on "similar" forms (e.g., supposed symptomatic focal epilepsies in children or adults) should be performed to confirm this hypothesis.

In the context of normal brain development, this pattern suggests either a fixed deviation from the normal developmental template or a delay in brain maturation. It was interesting to note that there is a structural reorganisation with redistributed nodes from the posterior head regions to more anterior frontal, and temporal regions, considering that the posterior cerebral areas develop before other areas when maturing from neonatal age to childhood, and to adulthood (Falsaperla et al. 2021). These altered brain topologies seemed to have adverse consequences, as these network configurations may be more predisposed to poorer cognitive performances and targeted attacks.
